# Is perinatal outcome of planned vaginal birth in multiple pregnancy influenced by using epidural analgesia: a systematic review

**DOI:** 10.1007/s00404-026-08476-2

**Published:** 2026-06-01

**Authors:** Ewout C. van der Wal, Johannes J. Duvekot, Ilse J. J Dons-Sinke, Robert J. Stolker, Sanne E. Hoeks, Caroline D. van der Marel

**Affiliations:** 1 Department of Anesthesiology, Erasmus MC Sophia, University Medical Center Rotterdam, P.O. Box 2040, 3000 CA Rotterdam, the Netherlands; 2 Department of Obstetrics and Gynecology, Division of Obstetrics and Prenatal Medicine, Erasmus MC Sophia, Rotterdam, the Netherlands

**Keywords:** Vaginal delivery, Epidural anesthesia, Multiple pregnancy, Twins, perinatal outcomes

## Abstract

**Purpose:**

Epidural analgesia (EA) is considered safe and advantageous in twin deliveries, yet specific evidence to support this practice is scarce. This systematic review aims to identify the impact of EA on perinatal outcomes during planned vaginal twin deliveries.

**Methods:**

A literature search was conducted to identify studies involving vaginal multiple delivery with EA. Newcastle–Ottawa scale quality assessments were performed, and baseline characteristics and perinatal outcomes were extracted.

**Results:**

Seven retrospective cohort studies were included. Quality scores ranged from 5 to 8/8 stars. Baseline characteristics and reported outcome measures were heterogenous. When comparing EA to non-EA users, incidence of CS after vaginal trial of labor was similar in one study (13% vs 21%, OR 1.65 [0.70–3.90]), but lower in another study (7% vs 15%, OR 0.0435 [0.022–0.083]). A third study reported a beneficial effect of EA for the second twin only. Anesthesiologic strategies for emergency CS were not reported. Instrumental delivery was increased or similar for women in the EA group. Apgar scores for deliveries using EA were not reduced. Perinatal mortality for the second twin was similar.

**Conclusion:**

EA in planned vaginal twin delivery seems to be associated with a reduced incidence of CS, a higher risk of assisted delivery, and an increased second-stage length. However, evidence of the associated consequences for mothers and neonates is limited. Given the limited number of eligible studies and reported outcomes, further research is needed to definitively assess the current practice of routine epidural administration for multiple deliveries, and to correctly inform women of its risks/benefits.

**Supplementary Information:**

The online version contains supplementary material available at 10.1007/s00404-026-08476-2.

## Introduction

Neuraxial analgesia is considered the gold standard for maternal pain relief during vaginal delivery. In the absence of contra-indications, a neuraxial block may freely be provided to mothers requesting analgesia [1]. The most frequently used method of neuraxial blockade in obstetric anesthesia is epidural analgesia (EA) [2, 3].

EA is associated with specific advantages and disadvantages. Advantages include positive physiological and psychological effects, mainly through pain relief and stress reduction [4–7]. Disadvantages of EA include potential inherent side effects, such as hypotension, pruritus, urinary retention, and motor block. Potential complications include local anesthesia toxicity, high neuraxial block, postdural puncture headache, pneumocephalus, spinal epidural hematoma, and infection [8–10]. The number of instrumental deliveries used to be slightly higher in parturients receiving EA, but in more recent studies no difference was found. This is probably due to lower doses of local anesthetic being used, reducing motor block and maternal ability to push. EA does not affect the neonate [11]. However, these studies are all based on single-child deliveries.

Specifically in multiple pregnancy, EA is considered advantageous as a risk mitigation tool. Twin delivery is associated with a higher chance of assisted delivery and conversion to emergency cesarean section (CS) [12–14]. Neuraxial anesthesia avoids the need for general anesthesia, which yields a higher risk for complications in pregnancy [15–17]. An epidural catheter inserted at the onset of labor also mitigates the need for placement during emergency situations, which may be more complicated than elective placement and may cause delays in time-critical situations.

Apart from providing sufficient analgesia, the goal of recommending EA in vaginal twin deliveries is to create safe (anesthetic) conditions. Even though this seems advantageous and the practice is generally deemed safe in this population [17], the literature providing evidence to support this practice is scarce. As a consequence, current guidelines rely heavily on professional consensus. Therefore, a systematic review on the effect of EA in vaginal twin deliveries was deemed necessary.

Regarding EA for vaginal delivery, several important outcomes are to be considered. Since general anesthesia for emergency CS is associated with significantly increased morbidity and mortality, its incidence is considered the most significant primary outcome [18]. Significant secondary outcomes include the need for instrumental and/or surgical delivery, duration of labor, and Apgar scores.

This article systematically reviews studies evaluating the perinatal outcomes for both mothers and neonates during planned vaginal twin deliveries, comparing EA with non-EA.

## Methods

This systematic review was registered in PROSPERO (CRD42021256481) and adheres to the Preferred Reporting Items for Systematic Reviews and Meta-Analyses (PRISMA) statement [19].

The following databases were included in the systematic literature search: Embase, MEDLINE, CINAHL, Web of Science and Cochrane central. The full search strategy can be found in Appendix 1. Reference manager Endnote was used to remove duplicates and screen articles [20]. Language restrictions were not applied, and studies from all sources were considered. All titles and abstracts were independently screened by two authors (EW, CM), and a selection was made based on the eligibility criteria. The selected articles were then screened again in full-text, and a final selection was made. Disagreement was resolved by consensus.

Included were randomized controlled trials and cohort studies describing the use of EA during labor in multiple pregnancies with planned vaginal deliveries, irrespective of gestational age and the number of neonates. Excluded were case-reports and case-series, abstracts, letters, and articles without information on perinatal outcomes. Articles describing planned deliveries through CS with spinal and/or combined spinal epidural anesthesia were also excluded. Articles published in 1990 or before were also excluded, due to major changes in obstetric and anesthesiologic practice since then. Articles exclusively describing trial of labor after cesarean delivery were not included: this is a very specific group of parturients whose outcomes cannot be extrapolated to regular twin deliveries.

The following data were extracted from the eligible reports: first author, year of publication, amount of included patients, number of patients with and without EA, epidural catheter and medication characteristics, maternal age, parity, maternal height, gestational age at labor, labor induction, fetal presentation, labor duration, mode(s) of delivery, birth weight, Apgar scores, and perinatal mortality.

Since all included reports were cohort studies, their quality was assessed by means of the Newcastle-Ottawa Scale [21]. This tool assesses methodological quality of participant selection, comparability of study groups, and reported outcomes. Due to the expected small number of eligible studies, no quality threshold was included in the eligibility criteria.

Study details (including baseline characteristics), outcome measures, and outcomes were reported in separate tables.

Based on reported measures per outcome and reported outcomes per study, eligible outcomes were selected for incorporation into a meta-analysis. Outcomes were considered eligible when at least three studies reported similar outcome measures (including measures of precision where needed).

## Results

**Fig. 1:**
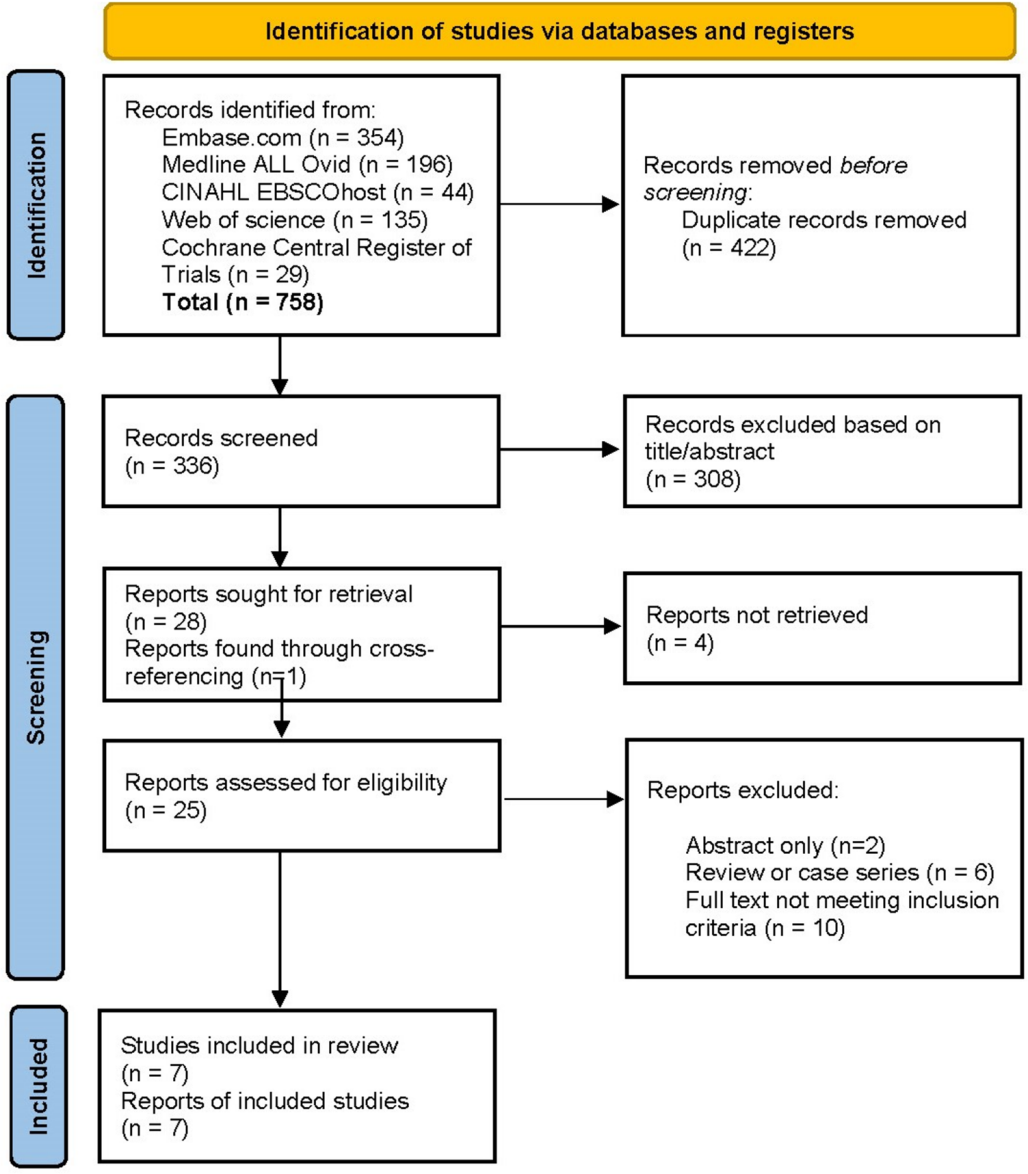
Flowchart of the inclusion and exclusion process

The literature search identified 758 articles (April 2, 2024). Figure 1 shows a flowchart of the inclusion and exclusion process. After duplicate removal, 336 articles were screened based on title and abstract. 308 were excluded based on the eligibility criteria and four were not retrievable, resulting in a total of 28 articles eligible for full-text screening. One additional report was found through cross-referencing. 22 articles were then excluded based on their full texts, six of which met most inclusion criteria, but dated from 1975 until 1987. Eventually, seven reports were eligible for systematic review [22–28].

Study characteristics for the included reports are shown in Table 1. All selected studies are retrospective cohort studies. Inclusion criteria, such as gestational age, mode of delivery, and fetal presentation, were not always described and varied greatly per study, for both intervention and control groups. Methods of EA administration, if described, varied with bupivacaine dosages ranging from 0.1% to 0.25%, sometimes combined with fentanyl. No additional epidural characteristics were reported in any of the studies. Control group interventions were the administration of either remifentanil PCA, meperidine, or nitrous oxide. None of the studies incorporated delivery of more than two neonates into their analyses. One study only reported second-born outcomes, after vaginal delivery of the first twin [28]. Four studies excluded preterm deliveries (gestational age < 35 weeks) [24–27].

**Table 1:** Study characteristics

Study	Type of study	Population	Epidural group characteristics	Control group characteristics	Total included women (n)	Epidural group (n)	Control group (n)	Epidural catheter and medication characteristics	Control group interventions
Peretz (2019)	Retrospective cohort	Twin pregnancies, > 24 weeks, vaginal delivery	Maternal request	Maternal request	827	332	495	**1995 to 2005**: 0,25% bupivacaine 10 ml intermittent bolus **2005 to 2015**: 0,125% bupivacaine with 2 mcg/ml PCA	Meperidine, nitrous oxide
Rottenstreich (2020)	Retrospective cohort	Vaginally delivered twin fetuses, gestational age > 34 weeks	n/a^a^	n/a	1751^1^	n/a	n/a	n/r	n/a
Farghali (2021)	Retrospective cohort	Twin pregnancies, > 34 weeks, vertex presentations, no induced labor, vaginal delivery	All women except: patient refusal, active maternal hemorrhage, septicemia, infection at or near the site of needle insertion and clinical signs of coagulopathy	Women with contraindications to epidural analgesia	343	231	112	Bupivacaine 0.125% + fentanyl 1 µg/Ml, 6-10ml/hour	Remifentanil PCA
Levin (2021)	Retrospective cohort	Twin deliveries of healthy twins, excluding operative delivery, surgical delivery, or nonvertex presentation of the first twin	n/r	n/a	2009	1625 (579 nulliparous, 974 parous, 72 grand multiparous)	384 (76 nulliparous, 261 parous, 47 grand multiparous)	n/r	n/r
Zidan (2021)	Retrospective cohort	Twin pregnancies, > 34 weeks, vaginal delivery, reaching second stage of labor	Regional anesthesia offered to all women with twin pregnancies, and recommended in scenarios, such as non-cephalic second twin	n/a	345	311	34	Walking epidurals not routinely provided	n/r
Lucovnik (2023)	Retrospective cohort	Vaginal twin deliveries ≥ 34 weeks	Maternal request	Maternal request	244	40	204	Bupivacaine 0.1% + fentanyl 2 µg/mL, hourly programmed intermittent epidural bolus of 5–10 mL + PCEA 5 mL bolus, 10 min lockout	Remifentanil PCA
Vaajala (2023)	Retrospective cohort	Twin deliveries, first twin vaginal	n/r	n/r	6022	3242	2780	n/r	n/r

*n/a* not applicable

*n/r* not reported

^a^In this study, the population consists of neonates (instead of women), delivered through vacuum extraction

Baseline characteristics per study are reported in Table 2. There is high inter-study variability regarding what baseline characteristics were reported, and how they were reported. Maternal age was reported in all studies, with means ranging from 29.0 to 33.3 years. Nulliparity ranged from 20 to 90% (6 studies). Gestational age was reported in all studies, but in different manners (mean or intervals). Induction of labor ranged from 8 to 78% (all studies). Fetal presentation for both twins was reported only in one study [26].

**Table 2:** Baseline characteristics

Study	Total included women *(n)*	Maternal age *(years)*	Nulliparity	Gestational age *(weeks)*	Induction of labor	Birth weight twin 1: *(grams)*	Birth weight twin 2: *(grams)*	Fetal presentation *(twin1/twin2)*
Epidural group – Control group								
Peretz (2019)	827	29 (26-33) - 30 (26-34)	166 (50%)* - 135 (27%)*	37.0 (35.6–38.3)* - 36.6 (34.3–38.0)*	53 (16%)* - 39 (8%)*	2538 (2184–2842)* – 2365 (2000–2700)*	2409 (2105–2784)* – 2390 (1930–2685)*	(n.r./vertex): 541 (65%) (n.r./breech): 176 (21%) (n.r./transverse): 97 (12%) (n.r./unidentified): 13 (2%)
Lucovnik (2023)	244	31.5±4.7SD - 32.3±5.0SD	36 (90%) - 144 (71%)	35.9±1.04SD - 36.1±1.2SD	29 (78%) - 132 (65%)	2404±364 – 2543±352	2277±625 – 2466±401	(vertex/n.r.): 1625 (100%)
Vaajala (2023)	6022	30.1±5.0SD - 31.2±4.9SD	1717 (53%)* - 559 (20%)*	<32 wks: 105 (3.2%)* - 250 (9.0%)*	1840 (57%)* - 1278 (46%)*	- – -	- – -	-
Total group								
Rottenstreich (2020)	1751^a^	29.6±5.2SD	426 (24%)	37.2±1.5	205 (12%)	–	–	–
Farghali (2021)	343	V/V^b^: 31.6±5.7 C/C^b^: 29.8±5.4 V/C^b^: 31.3±4.7	–	V/V: 35.3±1.5 C/C: 35.8±1.7 V/C: 36.0±1.5	0 (0%)	–	–	(vertex/vertex): 343 (100%)
Levin (2021)	2009	31.20±5.22SD	655 (33%)	36.19±3.18SD	739 (45%)	- – -	- – -	(vertex/n.r.): 1625 (100%)
Zidan (2021)	345	30.49±5.00SD	130 (38%)	36.91±1.38SD	115 (33%)	- – -	- – -	-

*Marks a statistically significant difference

*n/a* not applicable

*n/r* not reported

^a^In this study, the population consists of neonates (instead of women), delivered through vacuum extraction

^b^*V/V* vaginal/vaginal, *C/C* cesarean/cesarean, *V/C* vaginal/cesarean

The risk-of-bias assessments using the Newcastle–Ottawa scale are shown in Table 3 [21]. Scores were at least five or higher on a scale of eight points, with lower scores indicating a higher risk of bias.

**Table 3:** Quality assessment

Cohort studies (Newcastle–Ottawa scale)									
	Selection				Comparability	Outcome			Score
First author	Representativeness of the exposed cohort	Selection of the non-exposed cohort	Ascertainment of exposure	Demonstration that the outcome of interest was not present at start of the study	Comparability of cohorts on the basis of the design or analysis	Assessment of outcome	Was follow-up long enough for outcomes to occur?	Adequacy of follow up of cohorts	Total score (out of 8)
Peretz (2019)	*	*	*	n/a	**	*	*	*	8
Rottenstreich (2020)	*	*	*	n/a	**	*	*	*	8
Farghali (2021)	*		*	n/a		*	*	*	5
Levin (2021)	*	*	*	n/a		*	*	*	6
Zidan (2021)	*	*	*	n/a		*	*	*	6
Lucovnik (2023)		*	*	n/a	*	*	*	*	7
Vaajala (2023)	*	*	*	n/a	**	*	*	*	8

Table 4 shows all study outcomes and conclusions per included study. For many outcomes, effect estimates and precision measurements (such as confidence intervals) were not provided in the reports. Regarding child-specific outcomes, some studies reported values for each neonate individually, while others only reported aggregated data for first- vs second-borns. Three or more studies lacked outcome reporting. This means that meta-analysis and other statistical analyses of outcomes, such as subgroup or sensitivity analysis, could not be performed.

**Table 4:** Outcomes per study

First author	Total included women	Women per study arm: Epidural group – Control group	*Maternal outcomes*				*Neonatal outcomes*			Study conclusion
			Mean labor duration: Epidural group – Control group *(hours)*	Mode of delivery twin 1: Epidural group – Control group	Mode of delivery twin 2: Epidural group – Control group	Mode of delivery twin 1 + 2: Epidural group – Control group	Apgar score at min. 5, twin 1: Epidural group – Control group	Apgar score at min. 5, twin 2: Epidural group – Control group	Perinatal mortality twin 2: Epidural group – Control group	
Peretz (2019)	827	332 – 495	- – -	Vacuum: 16 (5%) CS^*b*^: 67 (20%) – Vacuum: 19 (4%) CS: 39 (8%)	Vacuum: 16 (5%) CS: 67(20%) CS after first twin vaginal: 9 (3%)* – Vacuum: 16 (3%) CS: 39 (8%) CS after first twin vaginal: 38 (8%)*	- – -	**(< 7):** 1 (0%) – **(< 7):** 2 (0%)	**(< 7):** 1 (0%)* – **(< 7):** 12 (3%)*	- – -	Epidural analgesia in twin deliveries seems to be safe for the mother and the neonate, and minimizes the need for caesarean for the second twin
Rottenstreich (2020)	1751^*b*^	1539 – 212	- – -	- – -	- – -	Vaginal: 1320 (86%) Vacuum: 219 (14%)* – Vaginal: 206 (97%) Vacuum**:** 6 (3%)*	- – -	- – -	- – -	Epidural analgesia is a risk factor for vacuum delivery
Farghali (2021)	343	231 – 112	- – -	Vaginal: 208 (90%) CS: 23 (10%)* – Vaginal: 60 (79%) CS: 23 (21%)*	Vaginal: 194 (84%) CS: 37 (16%)* – Vaginal: 42 (16%) CS: 70 (63%)*	Vaginal: 402 (87%) CS: 60 (13%)* – Vaginal: 102 (46%) CS: 122 (54%)*	- – -	- – -	- – -	The use of epidural analgesia in twin pregnancies reduces the CS rate for delivery of both twins and reduces the rate of combined V/C for delivery of second twin
Levin (2021)	2009	1625: 579 nulliparous 974 multiparous 72 grand multiparous – 384: 76 nulliparous 261 multiparous, 47 grand multiparous	Second stage labor duration: Nulliparous: median 1.57* Parous: median 0.35* Grand multiparous: median 0.23* – Second stage labor duration: Nulliparous: median 0.75* Parous: median 0.17* Grand multiparous: median 0.12*	- – -	- – -	- – -	- – -	- – -	- – -	In women delivering twins, epidural use was associated with a statistically significant increase in length of the second stage
Zidan (2021)	345	311 – 34	Second stage labor duration: < 1h: 155 (50%) 1-2h: 69 (22%) > 2h: 87 (28%)* – Second stage labor duration: < 1h: 29 (85%) 1-2h: 3 (9% > 2h: 2 (6%)*	- – -	- – -	- – -	- – -	- – -	- – -	Use of epidural analgesia was an independent variable significantly associated with prolonged second stage
Lucovnik (2023)	244	40 – 204	- – **-**	- – **-**	- – **-**	**CS:** 5 (13%) – **CS:** 42 (21%)	- – -	** < 7:** 1 (3%) – ** < 7:** 3 (2%)	- – -	EA and remifentanil-PCA are safe and comparable in terms of labor outcomes in twin deliveries
Vaajala (2023)	6022	3242 – 2780	- – **-**	Inclusion only after vaginal delivery of first twin	Assisted vaginal: 1302 (40.2%)* CS: 64 (2.0%)* – Assisted vaginal: 823 (29.6%)* CS: 75 (2.7%)*	- – **-**	- – **-**	- – **-**	48 (1.5%)* – 81 (2.9%)*	Epidural labor analgesia was associated with a lower rate of emergency cesarean delivery and neonatal mortality for the se^1, 2^cond twin

*Marks a statistically significant difference

^a^*CS* cesarean section

^b^In this study, the population consists of neonates

### Primary outcome

Regarding mode of delivery, Lucovnik et al. describe the incidence of CS for the twin 1+ 2 group to be similar for EA vs no EA (13% vs 21%, OR 1.65 [0.70–3.90]) [27]. However, according to Farghali et al., women are at lower risk for CS for twin 1+ 2 in the EA group (7% vs 15%, OR 0.0435 [0.022–0.083], *p* < 0.0001). This study shows reduced CS incidence for twin 2 after twin 1 is delivered vaginally with EA (4% vs 5%, OR 0.380 [0.163–0.883], *p* < 0.001) [26]. Peretz et al. describe a similar increased risk for twin 2 after twin 1 is delivered vaginally (3% vs 8%, RR 0.29 [0.11–0.76], *p* = 0.01), but not for twin 1 (20% vs 8%, RR 2.81 [0.73–10.76], *p* = 0.13) [23].

### Secondary outcomes

Incidence of vacuum delivery was increased for the epidural group in both studies reporting this outcome, but the difference was only statistically significant in one study (Table 4).

Apgar scores were reported in two studies and were similar with and without EA for twin 1 (Table 4). For twin 2, Peretz et al. reported fewer reduced Apgar scores (< 7) for the EA group (0% vs 3%, adjusted *p* = 0.08), but Lucovnik et al. reported no statistically significant difference at all (3% vs 2%, OR 0.59 [0.06–5.51]) [23, 27].

Two studies reported second-stage labor duration as an outcome. The duration was increased for the epidural anesthesia group in both studies (Table 4).

Lastly, one included study reported perinatal mortality for twin 2, which was lower in the EA group (2% vs 3%, OR 0.61 [0.73–0.90]) [28].

## Discussion

The goal of this review was to summarize recent studies evaluating the influence of EA on perinatal outcomes during vaginal deliveries in multiple pregnancy.

The main finding of this study is that there is little recent evidence regarding EA in planned vaginal twin delivery. Current recommendations concerning EA in twin deliveries are based on limited evidence.

In modern medicine, it is important to use evidence to support common interventions, such as epidural analgesia, especially when there is a (albeit small) risk to the patient. Patient advocacy and informed consent are also becoming more important, so providers should be able to cite evidence to inform patients of the risks and benefits of epidural analgesia. Since existing evidence is based on single rather than multiple pregnancies, additional research is needed for this specific population. The aim is to gather higher level evidence to verify the use of EA in vaginal twin delivery.

This literature review identified seven eligible studies. Meta-analysis was impeded by the heterogeneous outcomes of the included studies and their lack of adequate method reporting, indicating that further research is required to come to definitive conclusions on EA outcomes for multiple deliveries.

Regarding our primary outcome, two studies report a lowered risk of emergency CS for EA users as compared to non-EA users [26, 28]. However, none of these studies report anesthesiologic strategies in case of conversion to CS.

In the event of an emergency CS, a previously inserted epidural catheter can be of great benefit, as it may help avoid general anesthesia with its increased risk for adverse outcomes and airway management problems, which may even result in maternal death. Therefore, not only is the risk of CS higher in the non-epidural group, but the consequences of conversion are potentially serious as well. These advantages are theoretical and seem to be of practical value for anesthesiologists. However, they are not supported by data from the papers included.

The remaining studies vary in their conclusions. Four studies conclude that the use of EA is safe and/or advantageous for both mother and child [26–29]. Overall, neonatal outcomes are sparsely reported even though they are among the most important factors to be regarded in obstetric research.

While three other studies report EA to be significantly associated with a prolonged second stage of labor or increased vacuum delivery, no resulting detrimental outcomes for mother or child were reported [22, 24, 25]. High concentrations of LA are associated with an inability to push effectively during the second stage of labor, which might explain these negative outcomes [30], but no anesthetic dosage information was provided in these three articles [22, 24, 25]. Dosages of ≤ 0.1% bupivacaine (or equivalent), as suggested by current guidelines, are associated with overall better outcomes during delivery, reducing motor blockade without reducing analgesia [30]. Therefore, we expect a lower incidence of assisted deliveries than reported in these studies when using currently advised LA dosages.

Our article is the first systematic review on this subject, even though EA during twin deliveries is widely used in obstetric practice. This illustrates the classical dilemma in obstetric anesthesia where clinical practice must rely on expert opinion due to the lack of recent evidence representing current practice in the literature. In this case, the practice of EA is based on clinical reasoning. Nonetheless, a situation where expert opinion is confirmed by high-level evidence would be preferable.

The limited number of eligible studies can be regarded as a weakness of this review. Alternatively, however, this can also be considered its most important outcome: this serves to underline the scarcity of research on the use of EA in planned vaginal twin deliveries, and the need for more data. This limited number is partly due to the exclusion of studies before 1990: over the course of several decades, daily practice in obstetric anesthesiology has changed extensively and older studies may be based on sub-par methodology. Therefore, evidence from before 1990 may not be as relevant to current practice. Another limiting factor is the sparse amount of measured neonatal outcomes in the included studies, especially for the first-born twin.

None of the included studies are randomized controlled trials, and only one included cohort study uses statistical adjustment of outcomes. (Retrospective) cohort studies on this subject are limited by confounding by indication and selection bias, and thus a significant bias may be present in these results. It is probable that EA is utilized earlier in women who are expected to have difficulties during labor, and, therefore, the outcomes for EA-users in cohort studies might artificially seem worse. This indicates the need for additional, well-designed trials. From an epidemiological point of view, we would recommend a randomized controlled trial on the use of EA in this population. However, this may be regarded as unethical, since many guidelines, based on older studies, already recommend EA. Furthermore, anesthesiologists should also be able to provide women with neuraxial anesthesia based on individual clinical indications and estimation of potential difficulties with airway management, and it should not be withheld from women requesting pain relief. An alternative may be a well-designed prospective cohort study.

A cohort study could also provide extra information on the influence of lower LA dosing, which enables modern practice of ‘walking epidurals’. Lower dosages are expected to have a positive influence on perinatal outcomes with EA, which was not considered in most studies included in this systematic review. In any case, trials on twin delivery would greatly benefit from more standardized reporting, to at least include the following: maternal characteristics, epidural characteristics (with LA concentrations), modes of delivery and Apgar scores for both neonates separately. Standardized reporting will aid in summarizing these outcomes into clinically relevant information, and thus help to provide the best possible twin delivery care in the future.

## Conclusion

Evidence on the influence of EA on twin delivery is scarce. EA in planned vaginal twin deliveries is reported to be safe. It is associated with a reduced incidence of CS, but is also associated with a higher risk of assisted deliveries and increased second-stage length. These conclusions are based on a limited number of studies with very few reported outcomes. Furthermore, none of the studies reported their anesthesiologic strategies for CS. Unfortunately for this systematic review we are bound by the low-quality evidence and, therefore, the extrapolated conclusions are not high-grade. This lack of research could be considered the most important outcome of this systematic review. The authors still think EA could freely be provided to women in labor, because of the important analgesic benefits. However, more high-level evidence is needed to provide information on the associated risks and benefits, to assess the current practice of routine use of EA in this patient population, and to be able to correctly inform patients.

## Supplementary Information

Below is the link to the electronic supplementary material.

Supplementary file1 (DOCX 14 KB)

## Data Availability

No datasets were generated or analysed during the current study.

## Abbreviations

CS: Cesarean section
EA: Epidural analgesia
LA: Local anesthetic
