# Supplementary material for: Is perinatal outcome of planned vaginal birth in multiple pregnancy influenced by using epidural analgesia: a systematic review
Source: Arch Gynecol Obstet. 2026 Jun 1;313(1):241. doi: 10.1007/s00404-026-08476-2 (PMC13437601; doi:10.1007/s00404-026-08476-2)
Supplement: Supplementary file 1 [file 404_2026_8476_MOESM1_ESM.docx]

**Appendix 1. Search strategy**

**embase.com**

('epidural anesthesia'/exp OR 'epidural analgesia'/de OR 'epidural catheter'/de OR 'epidural drug administration'/de OR (epidural* OR peridural* OR epi-dural* OR peri-dural*):ab,ti,kw) AND ('obstetric delivery'/exp OR birth/de OR 'cesarean section'/de OR 'obstetric patient'/de OR 'named groups by pregnancy'/exp OR pregnancy/exp OR childbirth/exp OR (delivery OR birth OR childbirth OR cesarean OR caesarean OR cesarian OR caesarian OR C-section* OR labor OR labour OR obstetric* OR parturient* OR pregnan*):ab,ti,kw) AND ('multiple birth offspring'/exp OR 'multiple pregnancy'/exp OR (multiplet* OR quadruplet* OR quintuplet* OR triplet* OR twin* OR multiple-pregnan*):ab,ti,kw) NOT (([Conference Abstract]/lim OR [Conference Review]/lim) AND [1800-2019]/py)

**Medline ALL Ovid**

(exp Anesthesia, Epidural / OR Analgesia, Epidural / OR (epidural* OR peridural* OR epi-dural* OR peri-dural*).ab,ti,kf.) AND (Delivery, Obstetric / OR Parturition / OR Cesarean Section / OR exp Pregnancy / OR (delivery OR birth OR childbirth OR cesarean OR caesarean OR cesarian OR caesarian OR C-section* OR labor OR labour OR obstetric* OR parturient* OR pregnan*).ab,ti,kf.) AND (exp Multiple Birth Offspring/ OR exp Pregnancy, Multiple/ OR (multiplet* OR quadruplet* OR quintuplet* OR triplet* OR twin* OR multiple-pregnan*).ab,ti,kf.) NOT ((news OR congres* OR abstract* OR book* OR chapter* OR dissertation abstract*).pt. AND 1800:2019.(sa_year).)

**CINAHL EBSCOhost**

(MH Anesthesia, Epidural + OR MH Analgesia, Epidural OR TI(epidural* OR peridural* OR epi-dural* OR peri-dural*) OR AB(epidural* OR peridural* OR epi-dural* OR peri-dural*)) AND (MH Delivery, Obstetric OR MH labor OR MH Cesarean Section OR MH Pregnancy + OR TI(delivery OR birth OR childbirth OR cesarean OR caesarean OR cesarian OR caesarian OR C-section* OR labor OR labour OR obstetric* OR parturient* OR pregnan*) OR AB(delivery OR birth OR childbirth OR cesarean OR caesarean OR cesarian OR caesarian OR C-section* OR labor OR labour OR obstetric* OR parturient* OR pregnan*)) AND (MH Multiple Offspring+ OR MH Pregnancy, Multiple+ OR TI(multiplet* OR quadruplet* OR quintuplet* OR triplet* OR twin* OR multiple-pregnan*) OR AB(multiplet* OR quadruplet* OR quintuplet* OR triplet* OR twin* OR multiple-pregnan*)) NOT ((MH News OR MH Abstracts OR MH Books+) AND PY 1800-2019)

**Web of science (SCI-EXPANDED & SSCI)**

TS=(((epidural* OR peridural* OR epi-dural* OR peri-dural*)) AND ((delivery OR birth OR childbirth OR cesarean OR caesarean OR cesarian OR caesarian OR C-section* OR labor OR labour OR obstetric* OR parturient* OR pregnan*)) AND ((multiplet* OR quadruplet* OR quintuplet* OR triplet* OR twin* OR multiple-pregnan*))) NOT (DT=(Meeting Abstract OR Meeting Summary) AND py=(1800-2019))

**Cochrane Central Register of Trials**

((epidural* OR peridural* OR epi NEXT dural* OR peri NEXT dural*):ab,ti) AND ((delivery OR birth OR childbirth OR cesarean OR caesarean OR cesarian OR caesarian OR C NEXT section* OR labor OR labour OR obstetric* OR parturient* OR pregnan*):ab,ti) AND ((multiplet* OR quadruplet* OR quintuplet* OR triplet* OR twin* OR multiple NEXT pregnan*):ab,ti) NOT "conference abstract":pt
